# Ureteric Calculus in a Left Complete Duplex System Masquerading as an Impacted Stone: A Case Report and Literature Review

**DOI:** 10.7759/cureus.41489

**Published:** 2023-07-07

**Authors:** Ayokunle Adenipekun, Nader Gaballa, Maitrey Darrad

**Affiliations:** 1 Urology, University Hospitals Birmingham NHS Foundation Trust, Birmingham, GBR; 2 Urology, University Hospitals Birmingham NHS Foundation Trust, Birmimgham, GBR

**Keywords:** endo urology, impacted stone, ureteroscopy, ctkub, duplex collecting system, ureteric calculus

## Abstract

Ureteric calculi are common urological problems. However, the management of stone disease varies considerably with patient choice and anatomy. There are few reports about ureteral calculi in patients with unilateral complete duplex kidneys managed by ureteroscopy. We hope to increase the awareness of urologists when investigating patients with similar symptoms.

We present the case of a 28-year-old man with recurring left flank pain. Non-contrast computed tomography (CT) scans of the kidney showed an impacted left vesicoureteric junction stone measuring 6 mm. Intra-operatively, a stone in the left duplex system was discovered. He was managed with ureteroscopy, laser fragmentation, and the insertion of ureteric stents.

The case demonstrates the complexity of managing ureteric stones in a complete duplex kidney. We highlighted the diagnostic limitations of non-contrast scans in identifying duplex systems. It is important to consider contrast CT scans in patients with unusually persistent symptoms to outline the anatomy of the kidney and collecting systems.

## Introduction

Renal duplication anomalies are common congenital urological conditions, affecting 1%-2% of the general population [1, 2]. Duplex kidney systems can be classified as complete or incomplete depending on the division of developing ureteric buds. They are either unilateral or bilateral, with unilateral duplication of the ureters being more common [2].

Duplex collecting systems are mostly asymptomatic and are classified as anatomical variants when incidentally discovered. However, duplex systems may be associated with ureteric obstruction, vesicoureteric reflux, urolithiasis, and subsequently renal scarring [3].

Lower ureteric stones are managed based on clinical symptoms, the size of the stone, and the anatomy of the patient. In general, stones less than 10 millimetres can be managed conservatively or with ureteroscopy (URS) or shockwave lithotripsy (SWL) [4].

We present a case report of a young man with a stone in a complete left duplex ureter, diagnosed as an impacted stone and managed with ureteroscopy, laser fragmentation, and stent insertion.

## Case presentation

A 28-year-old man presented on multiple occasions with persisting left flank pain. There was no fever or other symptoms suggestive of a systemic illness. He had a past medical history of ureteric stones. His observations and physical examination were normal at each presentation. Urinalysis revealed microscopic haematuria, while other blood biochemical parameters were normal (Table 1).

**Table 1:** The patient's serum biochemical parameters during different presentations to the hospital Reference range: Na (135–148 mmol/L); K (3.5–5.5 mmol/L); creatinine (60–120 mmol/L); urea (2.5–6.5 mmol/L); eGFR >90 Na: sodium; K: potassium; eGFR: estimated glomerular filtration rate; mmol/L: millimoles per litre

	Na (mmol/L)	K (mmol/L)	Creatinine (mmol/L)	Urea (mmol/L)	eGFR (ml/min/1.73m^2^)
March 2022	140	4.6	88	4.2	>90
May 2022	142	4.4	76	4.2	>90
Dec 2022	141	4.9	81	5.9	>90

**Figure 1:**
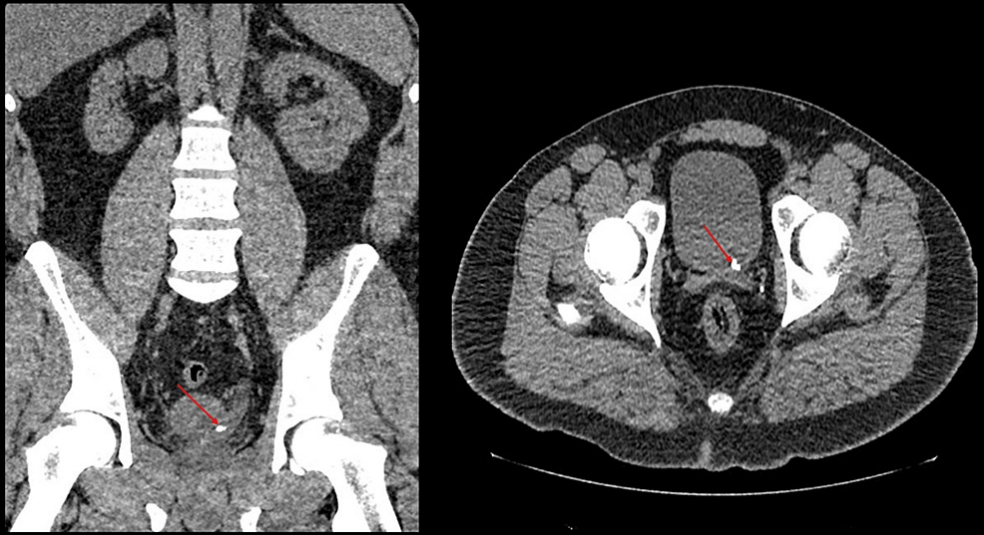
The first CTKUB shows the left VUJ stone measuring 6 mm. Left: coronal section; Right: axial view. The stone is labelled with red arrows. CTKUB: computed tomography of kidney, ureters and, bladder; VUJ: vesico-ureteric junction

**Figure 2:**
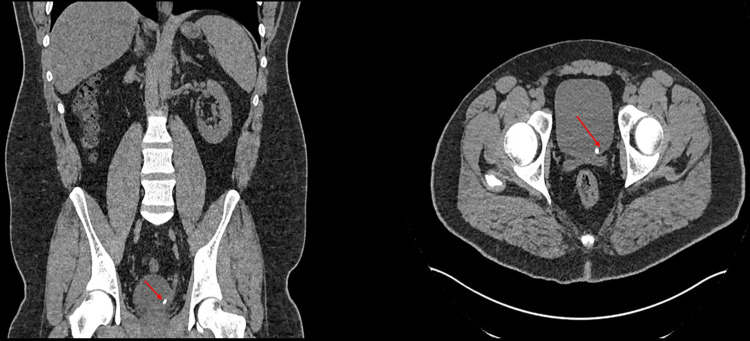
The second CTKUB, taken two months later, shows a persistent left VUJ stone measuring 6 mm. Left: coronal section; Right: axial view. The stone is labelled with red arrows. CTKUB: computed tomography of kidney, ureters and, bladder; VUJ: vesico-ureteric junction

He had serial non-contrast low-dose CT scans of the kidneys, ureters, and bladder (CTKUB), which showed a persistent left vesicoureteric junction (VUJ) stone measuring 6 mm with mild hydronephrosis. There were no other abnormalities or anatomical variants detected on non-contrast imaging (Figures 1-2). 

He was managed with analgesia and medical expulsive therapy using tamsulosin. Following the failure of conservative management, he was scheduled for a ureteroscopy and laser fragmentation of the stone.

Intra-operatively, the initial cystoscopy was normal, and both single left and right ureteric orifices were seen. The left ureteroscopy did not reveal the stone previously seen on imaging. However, on the table, fluoroscopy demonstrated a definite radio-opaque shadow medial to the normal left ureteric orifice. This radio-opaque shadow corresponded to the same left VUJ stone on the previous CTKUB, but this wasn’t visible on the endoscopy. On withdrawal of the ureteroscope out of the ureter, a bladder bulge was noticed medially to the normal left ureteric orifice with a suspected stone inside. This bulge was in the area corresponding with the shadow seen on fluoroscopic imaging.

**Figure 3:**
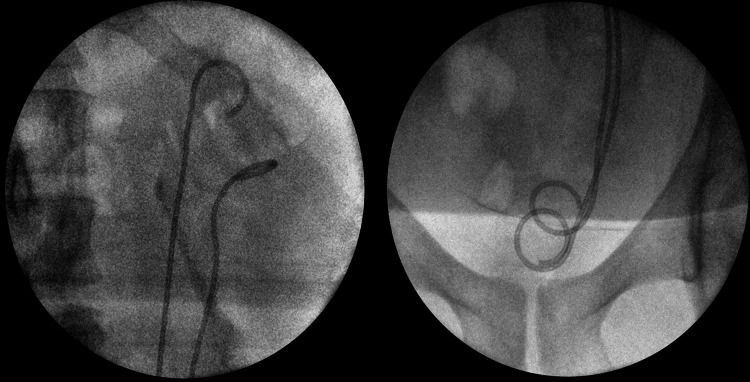
Fluoroscopy shows complete left duplex ureters, outlined by double-J ureteral stents. (Left: upper ureters; Right: lower ureters)

A decision was made to laser incise at the roof of the bladder bulge, revealing a stone. On identification and extraction of the calculus, a second left ureteric opening, medial to the normal ureteric opening, became apparent. This 6 mm stone was seen at the distal portion of the upper ureteric moiety. No further ureteric stones or abnormalities were identified on a complete ureteroscopy of the upper ureter. A retrograde fluoroscopic examination confirmed complete left ureteral duplication. Two double-J ureteric stents were inserted into the ureters at the end of the procedure (Figure 3).

Follow-up imaging has shown stone-free status, and both stents were removed in the clinic two weeks later. The patient remains asymptomatic.

## Discussion

Renal duplication anomalies are common congenital urological conditions, affecting 1%-2% of the general population [2]. The diagnosis is most commonly made via prenatal ultrasound, with hydronephrosis being the most prominent feature. The majority of these patients remain asymptomatic and do not require urological intervention [1, 5]. Similarly, this patient had no symptoms specifically arising from his complete duplex ureters. Urolithiasis is commonly seen in duplex systems and across several populations, with a wide-ranging prevalence between 4% and 20% [6]. Notably, in one of the CTKUBs, this patient also had an incidental right ureteric stone measuring 3 mm that passed spontaneously.

Embryologically, the ureters and collecting system of the kidneys are formed following the migration of a single ureteric bud at five weeks of gestational age. The arrival of the ureteric buds at the metanephros sets off a cascade that culminates in the development of nephrons and the collecting system [1]. Duplex ureters arise when there are abnormalities in the division of the ureteric bud from the Wolffian ducts. Complete duplex ureters occur when two distinct ureteric buds arise from the mesonephric duct and independently migrate to the metanephros, developing into two ureters: an upper ureteric moiety and a lower ureteric moiety. Although there are limited theories, abnormalities in the glial cell line-derived neurotrophic factor (GDNF)-RET signalling pathway have been implicated in the formation of duplex ureters [7, 8].

The lower ureteric orifice in this patient was located laterally and superiorly to that of the upper ureter. This anatomical relationship is well defined by Wiegert Meyer's rule, and it is typical of complete duplex systems. Occasionally, there are rare exceptions to this rule, as explained by the Stephen ectopic pathway [9]. Duplex ureters have unique complications associated with them due to their anatomy and relative position to each other. Ureterocoeles are most associated with the upper moiety ureter, given its long intravesical component, while vesicoureteric reflux, ureteropelvic obstructions causing pyelonephritis, and renal scarring are associated with the lower ureter [1]. The stone in this case was embedded in the intravesical portion of the upper ureteric moiety, necessitating a bladder mucosa incision to access the stone.

Ultrasonography, MRI, and CT urography are useful in making a diagnosis of complete duplex systems. Non-contrast CT scans are less likely to identify duplex ureters, especially without prior clinical suspicion [10]. However, a faceless renal appearance devoid of vascular or collecting system markings on a transverse CT section usually points to a duplex system [11]. Other investigations used in evaluating patients with duplex systems are voiding cystourethrography (VCUG) to check for vesicoureteric reflux and dimercaptosuccinic acid (DMSA) scans to assess scarring and function.

This patient had three CTKUBs with no report of a duplex system. A persistent stone at the left VUJ was consistently reported on serial imaging. Interestingly, his first CT scan reported a 6-mm VUJ stone with a 3-mm stone adjacent to it in the bladder. In retrospect, this 3-mm stone was in the lower ureteric moiety, further highlighting how easily duplex systems can be missed in non-contrast scans. This has important implications, especially in emergency cases where the wrong ureter may be stented, leaving the patient with an infected, obstructed system and sepsis requiring repeat imaging and procedures, as reported by H.H. Huang et al. [11].

The appropriate use of on-table fluoroscopy and identification of a radiopaque shadow in this patient were crucial in preventing a missed stone and subsequent complications. This is significant, considering there was an interval between the last CTKUB and the operation, thereby increasing the possibility of the stone being completely missed with the assumption that it had spontaneously passed. A recent positive CTKUB, fluoroscopic images, and close endoscopic examination all contributed to identifying this stone and duplex ureteric system in this case.

## Conclusions

This is a case of left complete duplex ureters with calculus diagnosed as an impacted stone. The case demonstrates the complexity of managing ureteric stones despite being very common. The diagnostic limitations of non-contrast scans in identifying duplex systems and the importance of on-table fluoroscopy were highlighted. The failure of conservative management necessitated surgical intervention in this patient. Importantly, a diagnosis of ureteric duplication should be considered in patients with persistent ureteric colic, recent positive imaging, and negative stones on initial ureteroscopy. Contrast CT imaging is useful to outline the anatomy and make a diagnosis when there is a suspicion of a duplex ureteric system. 
